# Supplementary material for: Sustained-input switches for transcription factors and microRNAs are central building blocks of eukaryotic gene circuits
Source: Genome Biol. 2013 Aug 23;14(8):R85. doi: 10.1186/gb-2013-14-8-r85 (PMC4054853; doi:10.1186/gb-2013-14-8-r85)
Supplement: Additional file 5 — HTML Browsable Motif Output. Zipped folder containing all WaRSwap and FANMOD motif output, viewable in a web browser. [file gb-2013-14-8-r85-S5.ZIP › HTML_browsable_motif_output/WaRSwap_all_tair9/sigs_WSWRSLDraw-2000_all.pvals.heatmaps.html/motif_id_238_011101110_tftype_all_upstream_-1000_0.html]

```
BG_MODEL = Rnew2
MOTIF_ID = 238_011101110
TF_TYPE = all
UPSTREAM = -1000_0


PVals
FN_0.2	FN_0.4	FN_0.6	FN_0.8
dg_60.genes	0.001	0	0.009	0.022
dg_70.genes	0.002	0	0.013	0.013
dg_80.genes	0.002	0.001	0.015	0.011

ZScores
FN_0.2	FN_0.4	FN_0.6	FN_0.8
dg_60.genes	3.215	3.864	3.208	-0.137
dg_70.genes	3.209	3.769	2.95	-0.117
dg_80.genes	3.144	3.753	2.947	-0.108

StDevs
FN_0.2	FN_0.4	FN_0.6	FN_0.8
dg_60.genes	1583.393	99.267	2.981	0.183
dg_70.genes	1598.866	100.765	3.2	0.115
dg_80.genes	1601.307	102.212	3.19	0.107
```
